# The complete chloroplast genome of *Dictamnus dasycarpus* Turcz. (Rutaceae)

**DOI:** 10.1080/23802359.2022.2109439

**Published:** 2022-08-22

**Authors:** Yan-ping Xing, Yue-yue Song, Yan-yun Yang, Che Bian, Wen-xiao Men, He-fei Xue, Liang Xu, Ting-guo Kang

**Affiliations:** School of Pharmacy, Liaoning University of Traditional Chinese Medicine, Dalian, China

**Keywords:** Chloroplast genome, *Dictamnus dasycarpus*, traditional Chinese medicine, phylogenetic tree

## Abstract

*Dictamnus dasycarpus* Turcz. 1842 is a medicinal plant of China. Its dry root bark is called BAIXIANPI, which is a common traditional Chinese medicine. Here, we report the complete chloroplast genome of *D. dasycarpus*. The length of the genome, large single-copy (LSC), small single-copy (SSC), inverted repeat (IR), and GC content was 157,056 bp, 84,497 bp, 18,487 bp, 27,036 bp, and 38.5%, respectively. A total of 132 genes were annotated, including 87 protein coding, eight rRNA, and 37 tRNA genes. Interestingly, 15 genes contained single intron while two others contained two introns. The phylogenetic tree showed the two *D. dasycarpus* (*D. albus*) clustered in a clade, which was sister to clade formed by the species of *Melicope*, *Tetradium*, *Phellodendron*, and *Zanthoxylum*.

*Dictamnus dasycarpus* Turcz. 1842 (Rutaceae) is an important medicinal plant in China. Its dry root bark called BAIXIANPI is an important traditional Chinese medicine, which is recorded in Chinese pharmacopoeia with the functions of clearing heat, drying dampness, expelling wind, and detoxification (Chinese Pharmacopoeia Commission 2020). In addition, it has certain curative effects on skin diseases; for example, the methanol extract of the BAIXIANPI has an anti-inflammatory effect and can be used to treat the imiquimod-induced psoriasis (Kim et al. 2013; Choi et al. 2019). Several active substances in BAIXIANPI have been reported such as limonoids, furoquinoline alkaloids, flavonoids, coumarins, sesquiterpenes, sesquiterpene glycosides, and phenolic glycosides (Souleles 1989; Chang et al. 2002; Nam et al. 2005). With such a high medicinal value, *D. dasycarpus* needs to be thoroughly studied in order to explore the underlying mechanisms and metabolic pathways. Here, we present the complete chloroplast genome of *D. dasycarpus*, which would provide basis for its molecular identification, genetics and breeding and protection of wild resources.

According to the Regulations of the People’s Republic of China on Wild Plants Protection, *D. dasycarpus* is not in the list of national key protection of wild plants. Article five of the regulations stipulates that the scientific research on wild plants and *in situ* and *ex situ* protection of wild plants are encouraged and supported.

This study was approved by the School of Pharmacy, Liaoning University of Traditional Chinese Medicine. All operations were carried out in compliance with the guidelines in Specification on Good Agriculture and Collection Practices for Medicinal Plants (GACP; number: T/CCCMHPIE 2.1-2018). Fresh leaves of *D. dasycarpus* were collected from Dalian, China (E 121°52′38.06″, N 39°03′46.59″). The voucher specimen (20210608003LY) was identified by Professor Tingguo Kang of Liaoning University of Traditional Chinese Medicine, and deposited in the herbarium of Liaoning University of Traditional Chinese Medicine (Yanping Xing, 46730131@qq.com). The genomic DNA was stored in the Key Laboratory of Traditional Chinese Medicine in the University (Dalian, China) (Yanping Xing, 46730131@qq.com). In short, genomic DNA was extracted from leaf samples following previous protocols (Zhang et al. 2019) and sequenced on Illumina Hiseq (Illumina, San Diego, CA). NGS QC was employed to check sequencing data quality (Patel and Jain 2015). Using SPAdes, we assembled the chloroplast genome of *D. dasycarpus*, *de novo* (version 3.9.0). The annotation was conducted as previously described (Cui et al. 2019), and submitted to GenBank with the accession number MZ677241.

The chloroplast genome of *D. dasycarpus* was 157,056 bp in size, with a large single-copy region (LSC, 84,497 bp), a small single-copy region (SSC, 18,487 bp) and two inverted repeat regions (IRs, 27,036 bp). The GC content was 38.5%. Out of the 131 annotated genes, 87 were protein coding, eight were rRNA, and 37 were tRNA genes. Fifteen genes including *trn*K-UUU, *rps*16, *trn*G-UCC, *atp*F, *rpoC*1, *trn*L-UAA, *trn*V-UAC, *pet*B, *pet*D, *rpl*16, *rp*l2, *ndh*B, *trn*I-GAU, *trn*A-UGC and *ndh*A contained one, while *Clp*P and *ycf*3 contained two introns. The *rps*12 gene underwent trans-splicing.

Phylogenetic analysis was performed based on the complete chloroplast genome sequences of *D. dasycarpus* and 19 species obtained from GenBank that included 18 species of Rutaceae (including two samples from *Zanthoxylum bungeanum* Maxim. 1871 and a sample named *D. albus* L. 1753 a synonym for *D. dasycarpus*), and one species of Celastraceae (*Euonymus maackii* Rupr. 1857) as the outgroup. All the sequences were aligned by Mafft version 7 software (Katoh and Standley 2013) and phylogenetic analysis was conducted based on the maximum-likelihood (ML) implemented in IQ-TREE version iqtree-1.6.12 with a bootstrap of 1000 (Nguyen et al. 2015) and under the K3Pu + F+R6 model chosen according to the Bayesian information criterion (BIC). All the 17 nodes in the phylogenetic tree displayed a bootstrap value of 100. Figure 1 shows that the two *D. dasycarpus* (*D. albus*) clustered in a clade, which was sister to clade formed by *Phellodendron amurense* Rupr. 1857, *P. chinense* C. K. Schneid. 1907, *Tetradium daniellii* (Bennett) T. G. Hartley 1981, *T. ruticarpum* (A. Jussieu) T. G. Hartley 1981, *Z. asiaticum* (L.) Appelhans, Groppo & J.Wen 2018, *Z. bungeanum*, *Z. madagascariense* Baker 1889, *Z. motuoense* C. C. Huang 1978, *Z. pinnatum* (J.R.Forst. & G.Forst.) Druce 1917, *Z. tragodes* DC. 1824, and *Melicope pteleifolia* (Champion ex Bentham) T. G. Hartley 1993 (Figure 1).

**Figure 1.**
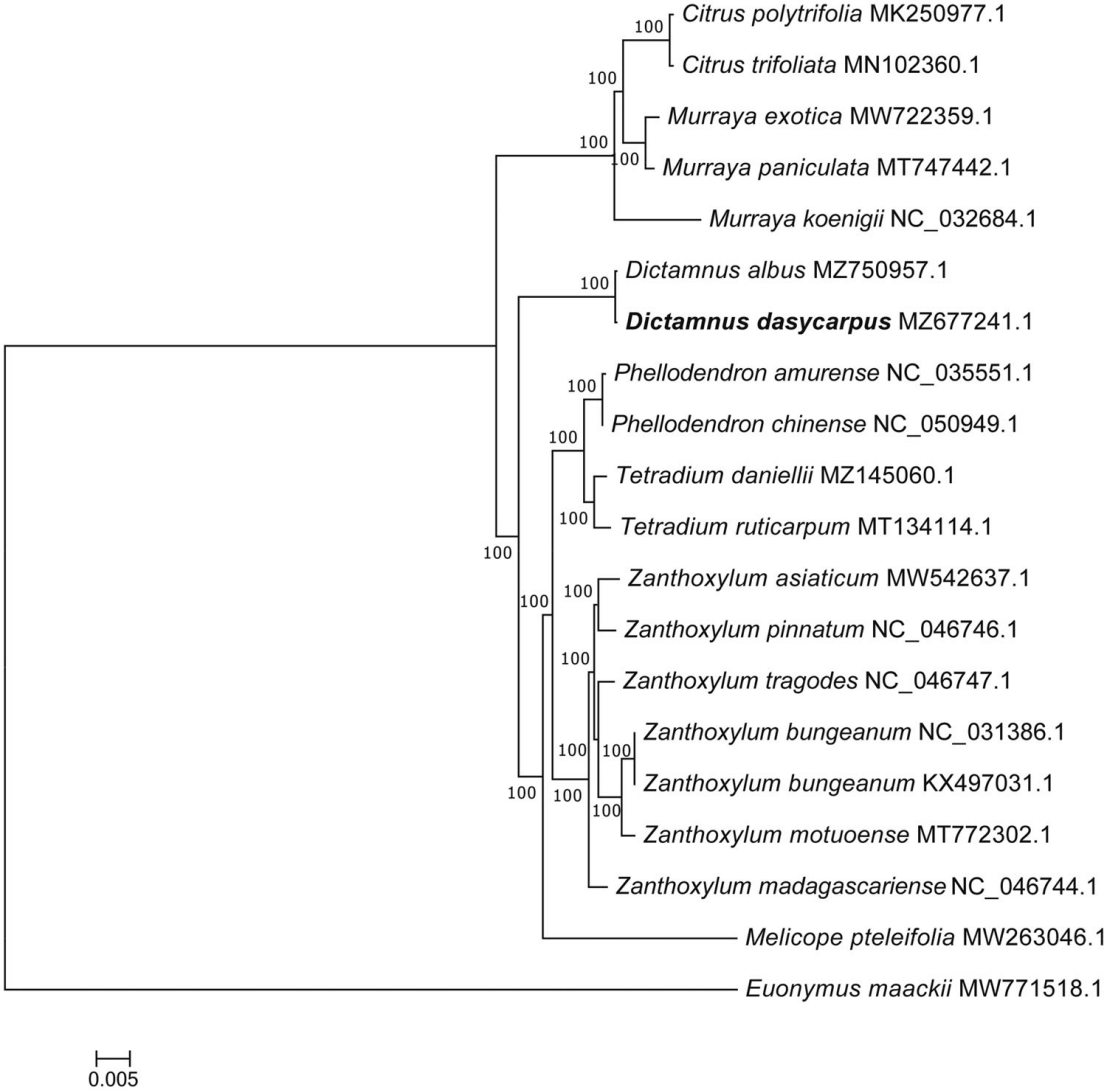
Phylogenetic tree construction of the 20 taxa by the maximum-likelihood (ML) methods based on the chloroplast genome sequences. The stability of each tree node was tested by bootstrap analysis using 1000 replicates. The *E. maackii* was set as the outgroup.

## Author contributions

Yan-ping Xing analyzed the data and drafted the manuscript; Yue-yue Song and Yan-yun Yang were involved in the analysis and interpretation of the data; Che Bian was involved in the acquisition of data, analysis, and interpretation; Wen-xiao Men and He-fei Xue were involved in the execution of the study; Liang Xu and Ting-guo Kang conceptualized and designed the study, and revised the manuscript critically for intellectual content and the final approval of the version to be published. All authors agree to be accountable for all aspects of the work.

## Data Availability

The genome sequence data that support the findings of this study are openly available in GenBank of NCBI at https://www.ncbi.nlm.nih.gov/nuccore/ under the accession no. MZ677241. The associated BioProject, SRA, and Bio-Sample numbers are PRJNA750882, SRX11628331, and SAMN20499492, respectively.
